# Broodstock development, induced spawning and larval rearing of the bilih, *Mystacoleucus padangensis* (Bleeker, 1852), a vulnerable species, and its potential as a new aquaculture candidate

**DOI:** 10.12688/f1000research.132013.2

**Published:** 2023-10-26

**Authors:** Hafrijal Syandri, Azrita Azrita, Rinold Thamrin, Deni Zen, Hendrik D. Roza, Jimmy Chandra Eduard Orah, Maman Abdurahman, Alif Yuza, Irvan Irvan, Afriwan Afriwan

**Affiliations:** 1 Department of Aquaculture, Faculty of Fisheries and Marine Science, Universitas Bung Hatta, Padang, West Sumatera, 25133, Indonesia; 2 Centre for Biodiversity Conservation, P.T. Semen Padang Indonesia, Padang, West Sumatera, 25237, Indonesia

**Keywords:** Mystacoleucus padangensis, Broodstock, Induced breeding, Hatching rate, Larval rearing

## Abstract

**Background:** *Mystacoleucus padangensis* living in Lake Singkarak, Indonesia, has high potential market demand but is threatened by overfishing and has not been successfully cultured. This study describes the first broodstock development, induced breeding, and larval rearing of *M. padangensis.*

**Methods:** A total of 1,000 female and 1,000 male broodfish were collected from the wild and reared in two concrete ponds (128 m ^2^) at the Centre for Biodiversity Conservation, P.T. Semen Padang, Indonesia. The broodfish were fed commercial feed to satiation at 09:00 and 17:00 h. The females (average weight 7.56 ± 0.85 g) and males (4.86 ± 1.20 g) were selected at a ratio of 1:4 (female:male), and gonad maturation was induced with a single dose of GnRH analogue (Ovaprim) of 0.1 ml/fish. At 16 h after hormone injection, eggs were collected individually into a plastic vessel. Spermatozoa were collected with sterile syringes. Eggs were fertilized using the "dry" method, and 0.5 ml samples (equal to 100 eggs) were taken. The eggs were incubated in a plastic strainer with a water volume of 1.57 litres and placed in a tarpaulin pond with a volume of 150.72 litres.

**Results:** The overall hatching rate was 78.93 ± 4.13%. The newly hatched larvae were 3900.81 µm long, with a yolk sac of 82881.480 µm ^2^. The mouth opened at 72 days post hatching (DPH) with a gape measuring approximately 61.880 µm. The protocol of larval feeding started with artificial feed, followed by Artemia nauplii up to 30 DPH. Weaning of larvae started at 4 DPH. Larvae started metamorphosis by 15 DPH and ended by 22 DPH when the larvae reached 7430.27 µm. Larval rearing resulted in an average survival rate of 28.4 ± 3.04%.

**Conclusions:** Its successful spawning induction and high larval hatching and survival rates make *M. padangensis* an excellent aquaculture candidate.

## Introduction

Diverse and unique fishery resources (including finfish, crustaceans, and molluscs) are increasingly recognised for their role in providing food security, improving nutrition, and ending malnutrition. ^1^^,^^2^ The availability of fish as a food source can help end hunger (United Nations Sustainable Development Goals, SDGs 2). ^3^ As defined by the Food and Agricultural Organization (FAO), food security encompasses many underlying factors in four key dimensions: ^4^ food availability, access, utilisation, and stability. Fish can contribute to these four dimensions because they are a source of micronutrients such as protein, lipids, minerals, and vitamins. ^5^^–^^8^ Additionally, fish are easy to harvest year-round and have low production costs. ^9^^,^^10^

Overall demand for fish has experienced a significant increase in recent decades. By 2018, the world registered a total fish production of 179 million tons (MT), of which 46% is from fish farming. ^11^ Of the total, 156 MT were used for human consumption, equivalent to an estimated annual supply of 20.5 kg per capita. ^11^ In the future, fish production from capture fisheries are expected to stagnate due to nonselective fishing. ^12^^–^^14^ Therefore, the four key dimensions of food security are not guaranteed. The only prospect to satisfy the world's fish demand is to improve its capacity for culturing in confined environments. ^15^

By 2018, Indonesia accounted for 6.1% of the world's aquaculture production. ^11^ Indonesia's total fish farming production was 16,032,122 metric tonnes (mt). A total of 3,374,924 mt (21.05%) was sourced from freshwater aquaculture production, 9,884,670 mt (61,65%) was sourced from marine aquaculture production, and 2,772,568 mt (17.29%%) was sourced from brackish water aquaculture production. ^16^ Over the past decade, the species of freshwater aquaculture commodities developed have included Nile tilapia, Clarias catfish, Pangasius catfish, common carp, and giant gourami, which have contributed 37.39%, 33.35%, 12.38%, 9.28%, and 6.98% of the total freshwater aquaculture production, respectively. ^16^ Efforts to encourage aquaculture development in Indonesia are constrained by the low number of successfully domesticated indigenous species that are aquaculture candidates. ^6^^,^^17^

A total of 1,300 fish species, including 40 endemic species, are recorded as living in freshwater in Indonesia. ^18^ *Mystacoleucus padangensis* (Bleeker, 1952) of the Cyprinidae family (Indonesian name bilih) is endemic to Lake Singkarak (the surface area is 112 km2), West Sumatera Province, Indonesia. ^19^^,^^20^

*M. padangensis*, a small Cyprinid, grows to approximately 20 cm and weighs 12 grams. ^21^ This fish species has the potential for high market demand. ^22^^,^^23^ From an economic perspective, around 700 fishermen depend on catching *M. padangensis*, ^24^ and the resource utilization rate of *M. padangensis* in Lake Singkarak reaches an average of around 94.53%. ^25^ However, this resource is threatened by excessive fishing and has not been successful in cultivation. ^26^^,^^27^ *M. padangensis* has been categorised as “vulnerable” by the International Union for Conservation of Nature (IUCN). ^28^ To determine the potential of *M. padangensis* as an aquaculture candidate in Indonesia, we (i) determined the reproductive characteristics of female and male broodstock reared under farm conditions in concrete ponds, (ii) induced spawning to obtain fertilisation and hatching rates in hatcheries, and (iii) investigated larval development of *M. padangensis* and survival up to 30 days after hatching with a protocol of pre-feeding with commercial feed and then with live food.

## Methods

### Ethical considerations

Captive *M. padangensis* broodfish were kept in concrete ponds in the Centre for Biodiversity P.T. Semen Padang, Indonesia to obtain data on female and male reproductive characteristics, induce spawning to obtain fertilisation and hatching rates, and determine the development of *M. padangensis* larvae up to 30 days after hatching. There were no required permits from the government of the Republic of Indonesia for this study. This research was funded by the Board of Directors of P.T. Semen Padang, Indonesia, under grant No. 0000072/HK.03.02/PJJ/50003897/3000/07.2022. This study received ethical approval from the Ethics Commission for Research and Community Service at Universitas Bung Hatta (092a/LPPM/Hatta/VII-2022). Ethical approval was given to collect fish samples and rear them in a pond, including induced spawning and larval rearing in hatcheries.

### Experimental animals

Lift nets in Lake Singkarak were used to catch 2,000 broodstock candidates of *M. padangensis.* These broodfish candidates were placed in an oxygenated polythene bag. The broodfish were transported by truck to the farm of Semen Padang Indonesia Resort, Padang City, West Sumatera Province. During the oocyte maturation stage, *M. padangensis* broodfish were reared under cultivation conditions. During oocyte maturational development, the broodfish were placed in two concrete ponds (8 × 8 × 2 m) separated by sex. The water level in each concrete pond was 1 m. Pond water was sourced from a reservoir owned by P.T. Semen Padang with a water inflow of 1 m ^3^ per second. During the rearing of broodfish, the water temperature and pH were between 24 and 26°C and 7.1 and 7.3, respectively. Dissolved oxygen varied from 6.4 to 6.8 mg/L, and alkalinity and hardness ranged from 65-68 mg/L HCO3 ^-^ and 52-55 mg/L CaCO3, respectively.

### Feeding

During domestication on the farm, the broodfish were fed commercial floating feed (Prima Feed L.P. 0, diameter size 1.6 mm, protein 33%, and lipid 4%). Feed is given three times a day and given to satiation at 09.00, 13.00, and 18.00. A total of 1,000 female and 1,000 male broodfish were reared in the concrete ponds. The average fish weight (FW) and the total length of the fish (FLT) of the twenty female broodfish were 7.56 ± 0.85 g and 9.31 ± 1.36 cm, respectively. At the same time, the average fish weight and total length of the twenty male broodfish were 4.86 ± 1.20 g and 6.38 ± 0.30 cm, respectively. Broodfish were analysed for the condition factor (CF), gonadal weight (GI), gonadal somatic index (GSI), absolute fecundity (AF), relative fecundity (RF), egg diameter (ED), semen volume (SM), semen pH (SpH), and motility of spermatozoa (MOL) from male broodfish.

The formula utilized for the analysis of the following parameters is as follows:

• CF = weight wet in gram/length ^3^ × 100
• GI = Fish gonadal weight is the size or total mass of fish reproductive organs called gonads
• GSI = (gonadal weight ÷ total body weight) × 100
• AF = is calculated by multiplying the number of eggs per 0.1 gram by the weight of the gonadal in grams
• RF = number of eggs in 0.1 gram of the gonadal weight

Sperm motility percentage (MOL) was determined by observing activated semen placed on a glass slide under a microscope, as described in the study by Azrita et al. ^6^ MOL examination was carried out using a digital microscope model EcoBlue with serial number S/N – EC 2203076, which was manufactured in Thailand. This microscope is equipped with an internal photo-imaging system connected to a computer for sperm motility examination.

The relationships between female brood weight and absolute fecundity and gonadal weight were assessed using the least square’s regression method. Similarly, the relationships between male broodfish weight within gonadal weight and semen volume were also assessed using the same method. Microsoft Office Professional Plus 2019 was used for plotting the figures.

### Assessment of oocyte maturational development and artificial induction of spawning

The female and male broodfish were checked for their gonadal maturity for spawning from early April 2022 onwards. Hence, to simplify catching broodstock, the pond water level was lowered to 20 cm, and then the broodstock was collected with a drive-in net with a suitable mesh size. Broodstock was fasted for 24 h before being captured. The broodstock is anesthetized orally with clove oil in 0.5 mL/10 L of water for ten minutes. The gonadal maturity stages (GMS) II, III, and IV of the female broodstock were classified as follows ^21^: in GMS II, the ovary containing the egg is dark green and visible to the naked eye; in GMS III, the ovaries fill approximately 70% of the abdominal cavity; eggs are greyish-green, visible to the naked eye, and larger than they are in GMS II; and in GMS IV, the ovaries fill approximately 80% of the abdominal cavity; visually, the ovaries contain greyish-green eggs that are larger and vary in size and the blood vessels are visible, especially in the ventrolateral area. Before hormone injection for ovulation, oocyte samples were taken from females *in vivo* as described previously ^21^ and were placed in Serra’s solution (6:1:1; 70% ethanol, 40% formaldehyde, and 99.5% acetic acid) for five minutes to determine the position of the cytoplasm. Then, the oocyte nucleus was classified using a four-stage scale as follows. ^29^ Stage 1: germinal vesicle in the central position, stage 2: early migration of the germinal vesicle (less than half of radius), stage 3: late migration of the germinal vesicle (more than half of radius), stage 4: germinal vesicle in the periphery or germinal vesicle breakdown (GVBD).

For ovulation and spermiation of broodfish, each female and male received one injection of GnRH analogue with a dopamine antagonist (Ovaprim trademark, fabricated by Syndel Laboratories Ltd, 2595 McCullough Rd. Nanaimo, B.C. V9S 4M9 Canada). The hormone was injected intraperitoneally in part of the left dorsal at 0.1 mL per fish. Based on the packaging, Ovaprim contains sGnRH-a (Salmon Gonadotropin-Releasing Hormone analog) as much as 20 μg/ml and domperidone as much as 10 mg/ml). These doses refer to the published amount for the ovulation of Cyprinids. ^30^ Fourteen to 16 h after hormone injection, broodstock ovulation and spermiation occurred. Eggs were collected individually into a plastic vessel and spermatozoa were collected with sterile syringes. Eggs were fertilised using the “dry” method, as described by. ^31^ The eggs were incubated in a plastic coconut milk strainer with a diameter of 20 cm and a water volume of 1.57 litres, and then moved to a circular tarpaulin pond with a diameter of 80 cm and a height of 45 cm that was filled with 150.72 litres of water. The water level in the tarpaulin pond was 20 cm, and the water temperature ranged between 24 and 26°C. Eggs were continuously aerated until they hatched. The eggs hatched after 19-20 h of incubation at 25 ± 1°C.

### Assessment of physicochemical parameters

The physicochemical values of temperature, dissolved oxygen, pH, nitrate-nitrogen, alkalinity, and hardness in circular tarpaulin ponds used for larval rearing of *M. padangensis* were noted every seven days. The water samples were collected at 10:00 am at a depth of 10 cm in each circular tarpaulin pond. A thermometer (Celsius scale) was used to measure water ( ^°^C) temperature, and an oxygen-meter (YSI Model 52, Yellow Instrument Co, Yellow Spring, OH USA) was used to measure dissolved oxygen (O2; mg/L). A digital pH-meter (Mini 0–14 pH I.Q., Scientific Cemo Science, Thailand) was used to determine the pH. The levels of nitrate-nitrogen (NO3-N; mg/L), alkalinity (mg/L), and hardness (mg/L) were analysed using standard methods for the examination of water and wastewater (APHA). ^32^

### Egg quality

Indicators of egg quality were estimated from the fertilisation and hatching rates of five spawning trials. The fertilisation rate was calculated eight hours after spawning and an estimated twelve hours before hatching. Fertilised eggs are transparent, while those that are not fertilised are opaque. Embryonic and larval development was examined under a digital microscope (EcoBlue, S/N – EC 2203076 Thailand) with a built-in photoimaging system connected to a computer for morphometric investigation. All larvae were fed artificial feed (BP Eguchi. Taiwan Co Ltd. Labelled 4% moisture, 42% protein, 34% fat, 7% ash, and 1% fibre) to apparent satiation three times a day, at 09.00, 13:00 and 18.00 h, from four days post-hatching (DPH) to 20 DPH. Live food such as *Artemia nauplii* was started by 17 DPH to 30 DPH. In this study, larval growth measured was total length every two days. The percentage of larval survival (%) during 30 days was measured as the number of metamorphosed individuals by the number of hatched-out larvae stocked multiplied by 100. The survival rate is expressed as the mean ± SD.

## Results

### Reproduction characteristics of *Mystacoleucus padangensis*

**Figure 1.**
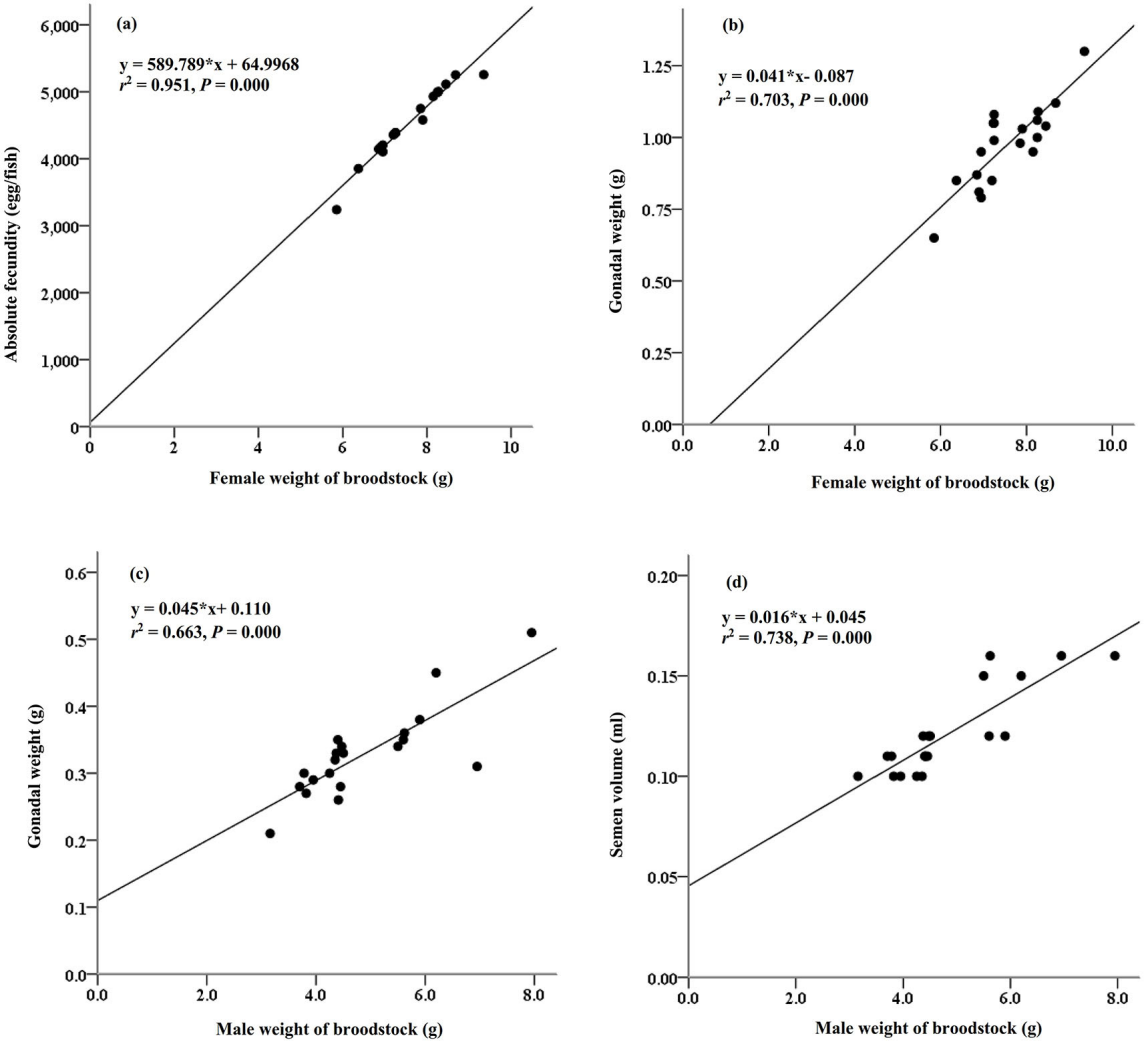
The relationships between the weight of the female bloodstock and absolute fecundity (a) and gonadal weight (b), and the relationships between the weight of the male broodstock and (c) gonadal weight (c) and semen volume (d).

The reproductive performance of *M. padangensis* females reared for 90 days is summarised in Table 1. The average live weight of the females was 7.56 ± 0.85 g, and the gonadal-somatic index (GSI) varied between 9.78 and 16.95%. Absolute fecundity varied between 3,539 and 5,656 eggs per female. The reproductive characteristics of the male fish are summarised in Table 2. The average live weight of the males was 4.86 ± 1.20 g, and the GSI and semen volume were 6.85 ± 0.83% and 0.12 ± 0.02 mL, respectively. The relationships between female brood weight and absolute fecundity (y = 589.789*×+64.997, *r* ^2^ = 95%, *P* > 0.000, Figure 1(a)) and gonadal weight (y = 0.041*×-0.087, *r* ^2^ = 70%, *P* = 0.000, Figure 1(b)) were determined, as were relationships between male sow weight and gonad weight (y = 0.045*×+ 0.110, *r* ^2^ = 66%, *P* = 0.000, Figure 1(c)) and semen volume (y = 0.016*× + 0.045, *r* ^2^ = 74%, *P* = 0.000, Figure 1(d)). The total number of female and male fish used to analyze these parameters was 20 individuals.

**Table 1.** Female size and egg characteristics of *M. padangensis* on the farm (mean ± SD, n= 20).

	Variable	Range (min-max)
FW (g)	7.56 ± 0.85	5.85–9.83
FLT (cm)	9.31 ± 1.36	7.23–12.5
CF	1.03 ± 0.40	0.37–1.94
GW (g)	0.97 ± 0.14	0.65–1.35
GSI (%)	12.88 ± 1.08	9.78–16.95
AF (egg/fish)	4,523 ± 515	3,539–5,656
RF (egg/g fish)	529 ± 109	393-816
ED (μm)	424.61± 7.59	413.35–444.5

Note: FW, fish weight; FL, fish length; CF, condition factor, GW, gonadal weight; GSI, gonadal somatic index; AF, absolute fecundity, RF, relative fecundity, ED, egg diameter.

**Table 2.** Male size and sperm characteristics of *M. padangensis* on the farm (mean ± SD, n = 20).

	Variables	Range (min-max)
FW (g)	4.86 ± 1.20	3.16–7.95
FLT (cm)	6.38 ± 0.30	5.9–70
CF	1.86 ± 0.43	1.15–3.17
GI (g)	0.33 ± 0.06	0.21–0.51
GSI (%)	6.85 ± 0.83	4.46–7.95
SV (ml)	0.12 ± 0.02	0.08–0.15
Semen pH	7.36 ± 0.17	7.1–7.6
Motility (%)	87.10 ± 2.44	83–90

Note: FW, fish weight; FL, fish length; CF, condition factor, GW, gonadal weight; GSI, gonadal somatic index; SV, semen volume; SpH, semen pH, MOL, motility.

**Figure 2.**
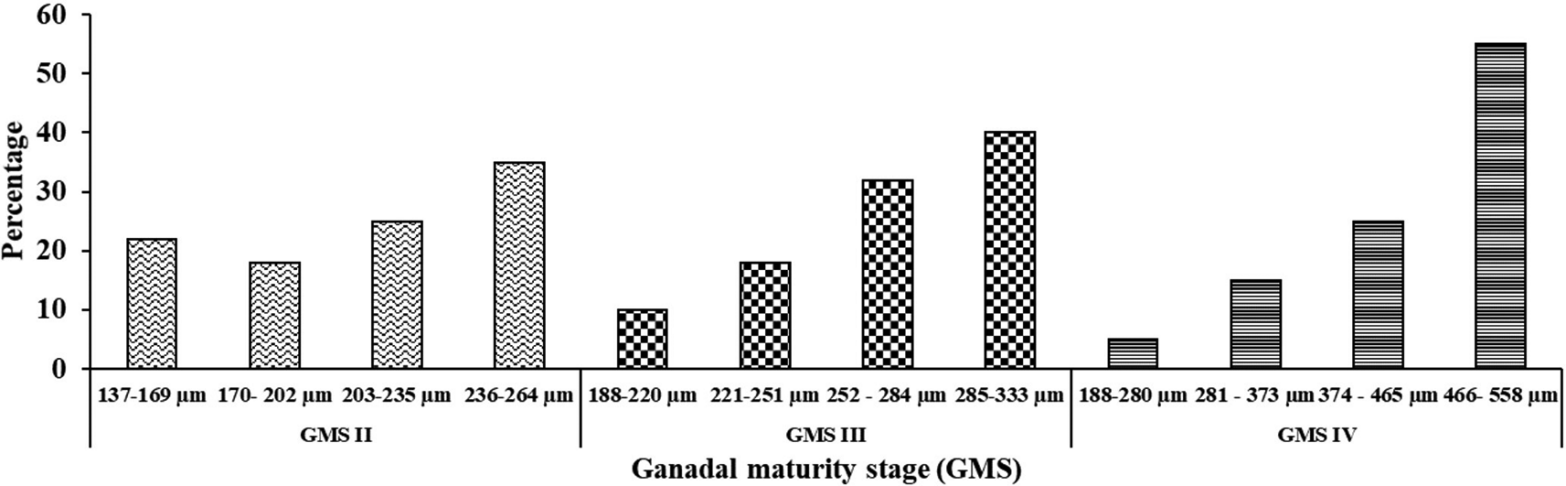
Distribution of egg diameter in GMS II, III, and IV.

The distribution of egg diameters in GMS II, III, and IV is shown in Figure 2. The maturation of female oocytes in *M. padangensis* is a synchronous batch, which means that the eggs mature simultaneously in a certain number. A group of eggs to be ovulated is found in development that is similar in terms of the size of each individual. At the GMS IV stage, most egg cells (around 55%) have reached gonadal maturity and are ready for fertilization, while only around 5% of the remaining eggs are still immature.

### Fertilisation and hatching rates

**Figure 3.**
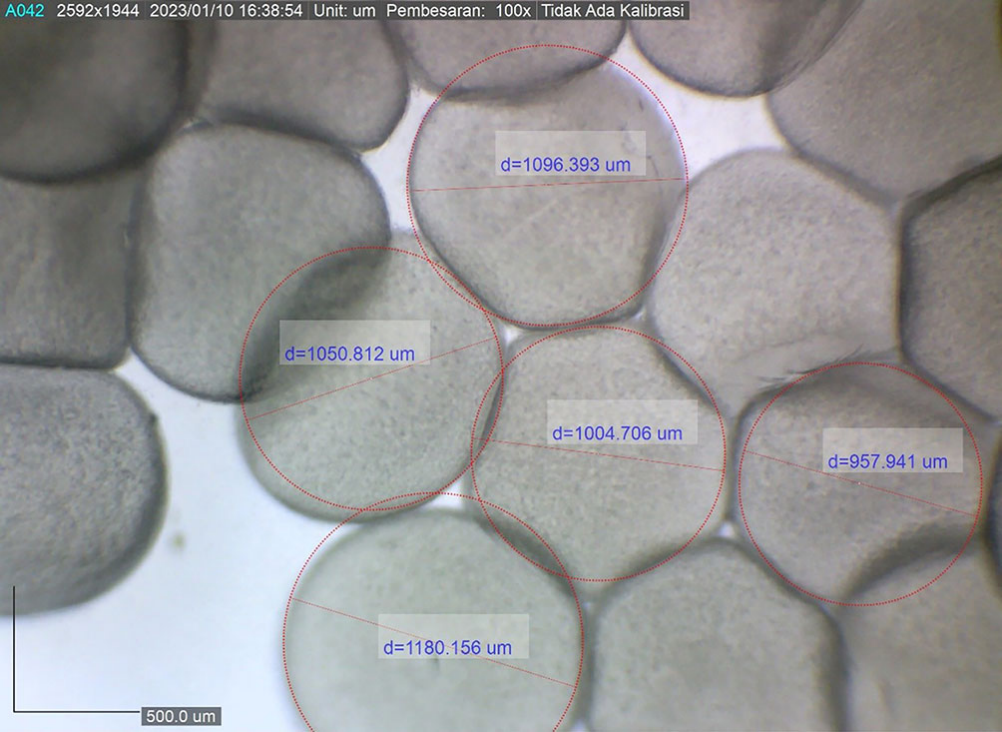
The egg diameter variation of eggs ovulated for spawning by *M. padangensis.*

**Figure 4.**
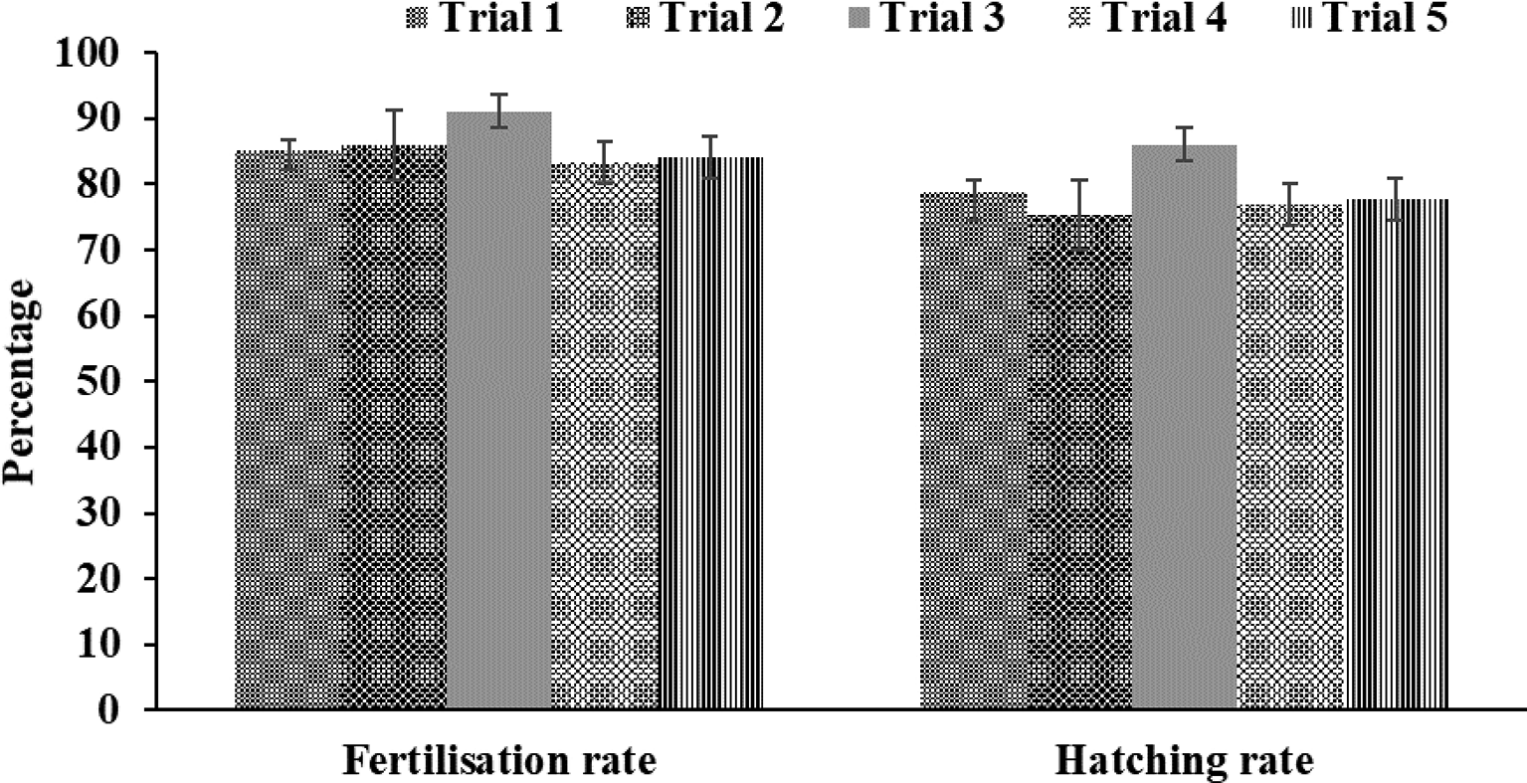
Fertilisation and hatching rates (%) of total collected eggs for five spawning trials.

**Figure 5.**
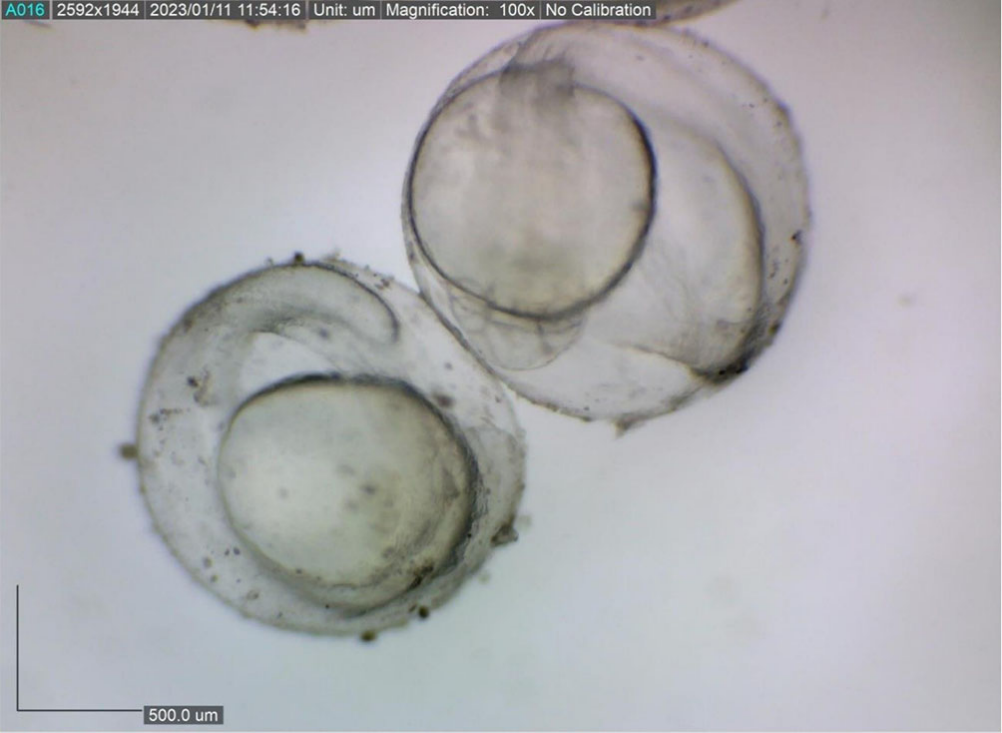
Hatching of eggs in progress after 15 hours of fertilization (Embryonic body formation and notocorda).

The present study showed that the size of the spawned eggs of *M. padangensis* before fertilisation was 957.941˗1,180.156 μm ( Figure 3). With a female and male sex ratio of 1:4, the fertilisation rate for five trials ranged between 83.33% and 91.11%, and the hatching rate varied from 77% to 86% ( Figure 4). Egg development occurred 15 h after fertilisation ( Figure 5), and the eggs hatched after 19-20 h of incubation at a water temperature of 25 ± 1°C. In this study, the water quality parameters in the larva reared in a circular tarpaulin pond during the experimental period included a water temperature varying from 24-26°C, dissolved oxygen ranging from 6.42-6.5 mg/L, and pH between 6.4-6.6. Additionally, alkalinity ranged from 51.5-53.5 mg/L HCO3-, hardness ranged from 64.5-66.5 mg/L CaCO3, and nitrite-nitrogen ranged from 0.02-0.05 mg/L. All physicochemical parameters were able to support embryo development and survival of the larvae.

**Figure 6.**
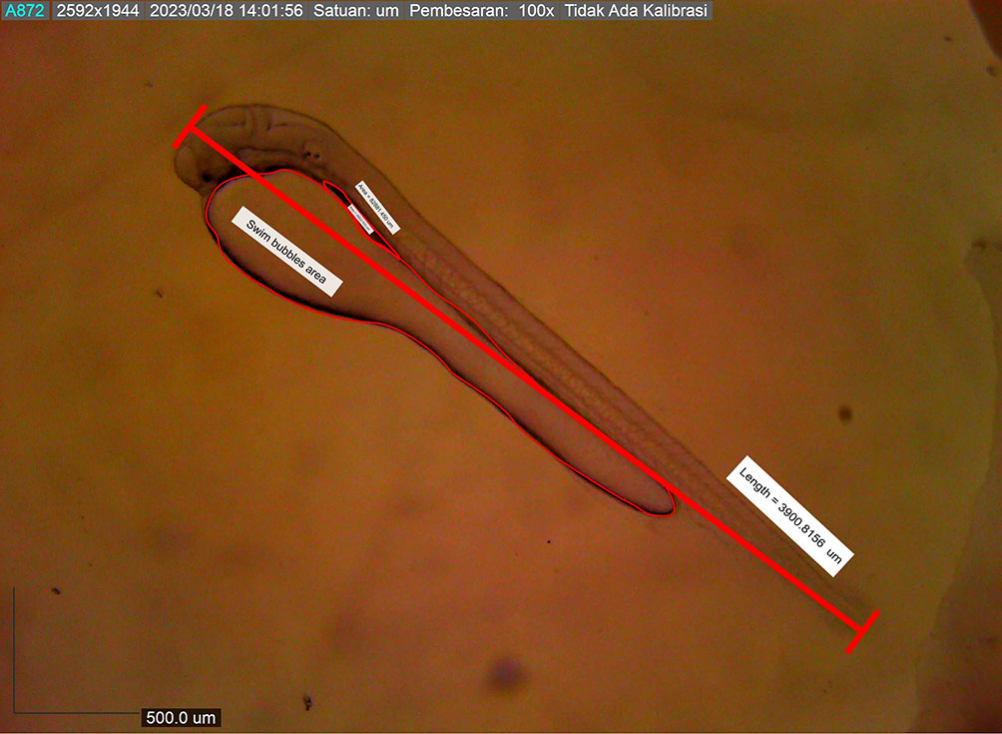
Newly hatched larva with nearly the exact measurements (3900.8156 ± 0.001 μm in total length).

**Figure 7.**
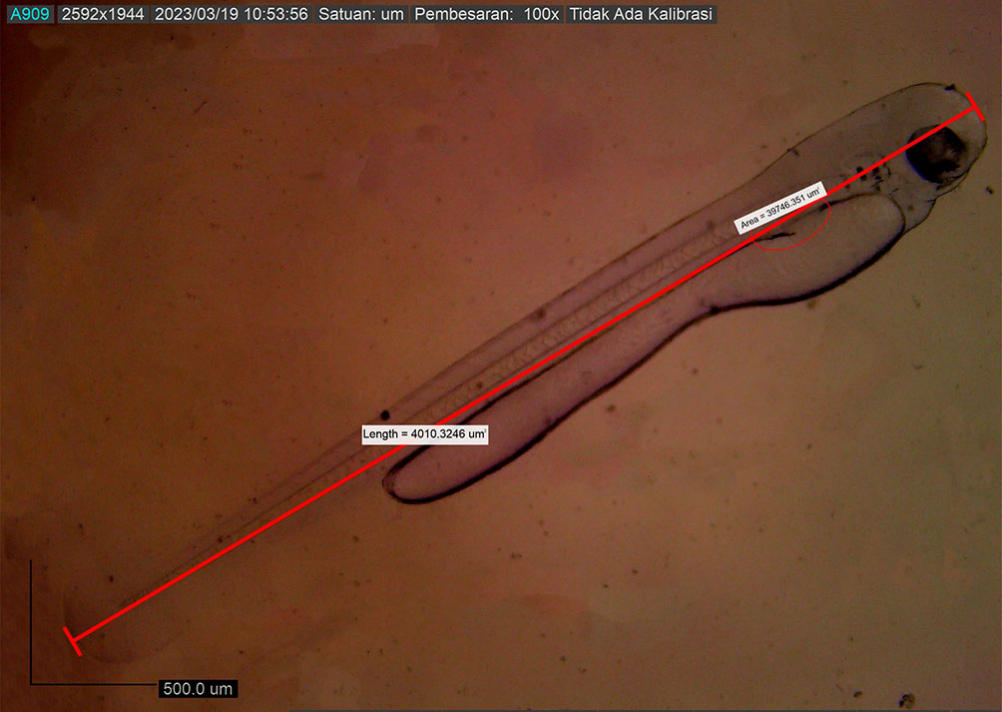
First-day posthatch larva showing the same measurements (4010.3246 ± 0.0011 μm in total length).

**Figure 8.**
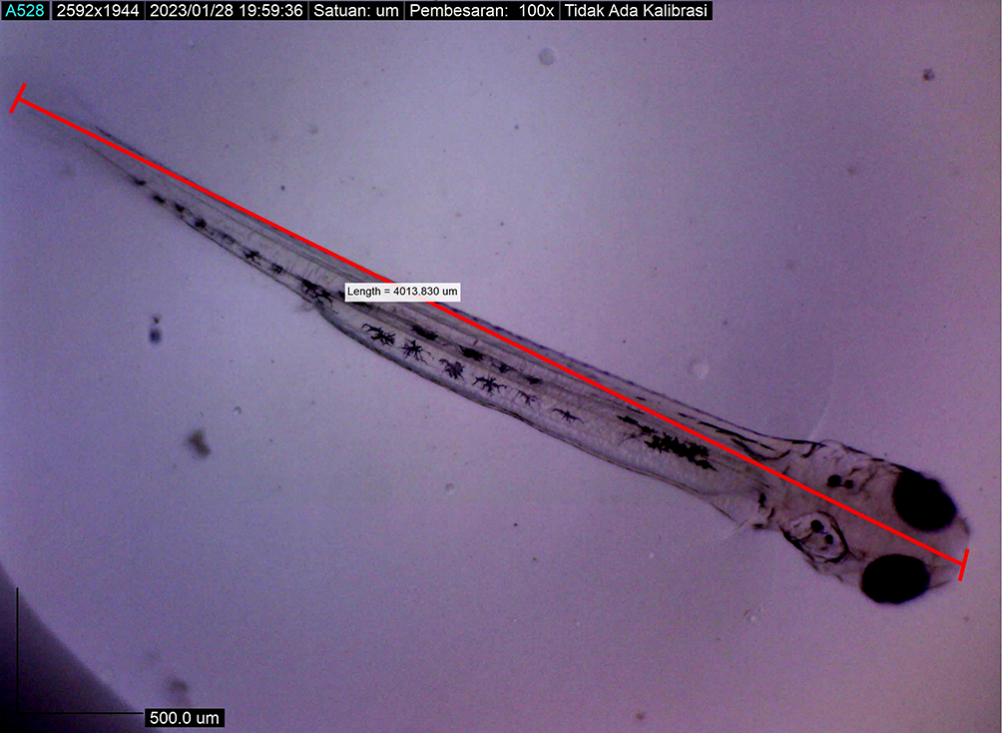
Larva 48 h after hatching, showing that their mouths had not yet opened (4013.8285 ± 0.0075 μm in total length).

**Figure 9.**
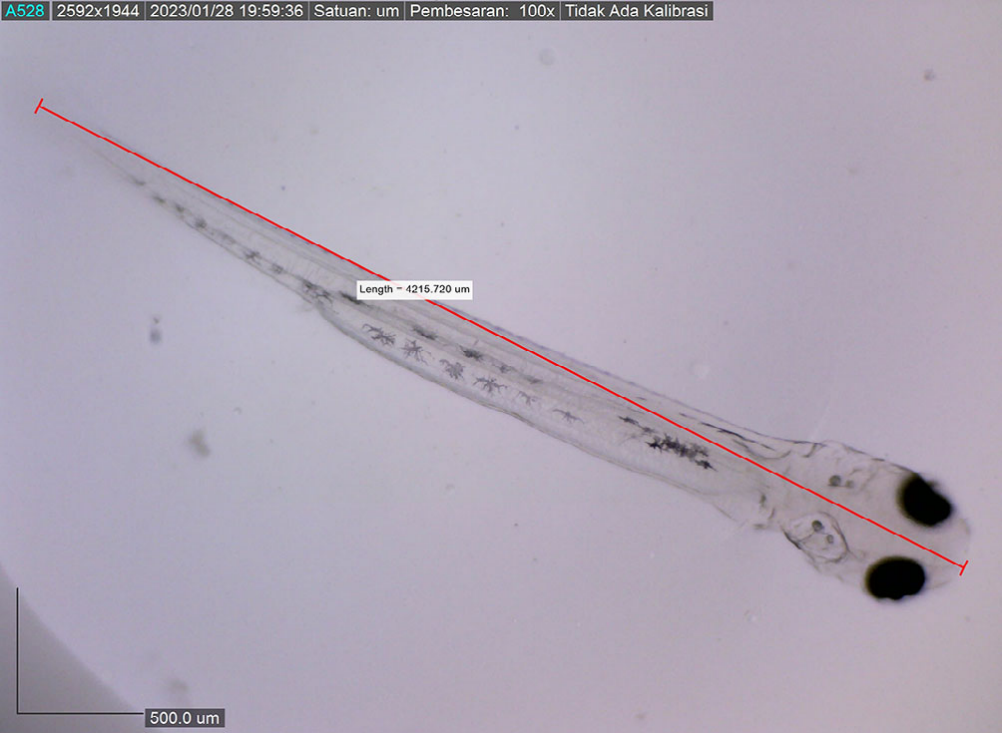
Larva 72 h post hatching, showing the mouth gape after mouth opening, with no yolk sac (4215.720 ± 0.0026 μm in total length).

**Figure 10.**
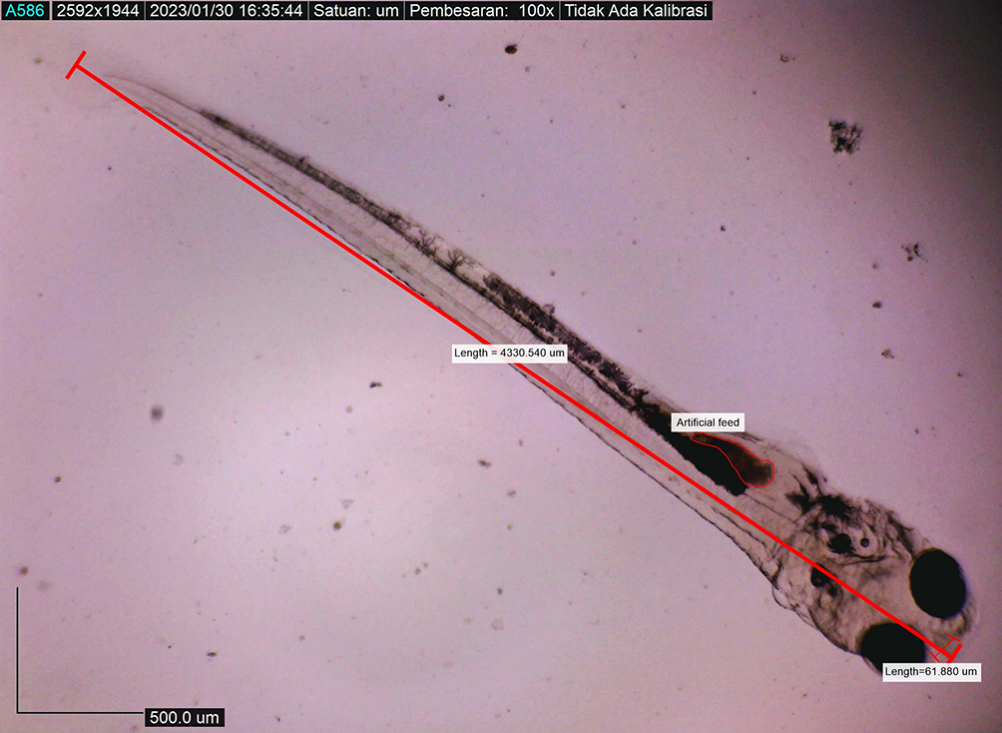
Fourth-day-post hatch larva showed gape mouth opening, and they ate artificial feed (BP Eguchi) to satiation (4330.4407 ± 0.0021 μm in total length).

Newly hatched *M. padangensis* larvae were seen swimming vertically to the surface of the water and returning to the bottom of the container. Then, larvae settled at the bottom of the tank and swam back vertically to the water’s surface. This movement was interpreted as a movement to fill the swim bubbles with air. Newly hatched larvae were 3900.8156 ± 0.001 μm in total length, with a slightly elongated oval yolk sac of 82881.480 μm ^2^ and 39822.933 μm ^2^ oil droplets in the area ( Figure 6). Body length on the first DPH was 4010.3246 ± 0.0011 μm, while the yolk sac decreased to 10214.043 mm ^2^ ( Figure 7). At 48 hours after hatching, the larval body length increased to 4013.8285 ± 0.0075 μm; the yolk sac was almost absorbed, and the eye began showing pigmentation with an eye diameter of 43.31 μm. At this stage, the appearance of the caudal fin and pectoral fins and the chromatophore pigmentation cells were black ( Figure 8). By 72 hours after hatching, the body length of the larvae increased to 4215.720 ± 0.0026 μm, the yolk sac was almost completely absorbed, and their mouths opened to 61.880 μm ( Figure 9). On the fourth day after hatching, larval body length was 4330.4407 ± 0.0021 μm, with an open mouth of 61.880 ± 0.0009 μm ( Figure 10). At that time, the artificial feed was given, namely, BP Eguchi, to satiation (BP Eguchi is an artificial feed formulated to increase shrimp fry growth). The size of the artificial feed was 31.716 ± 12.238 μm.

**Figure 11.**
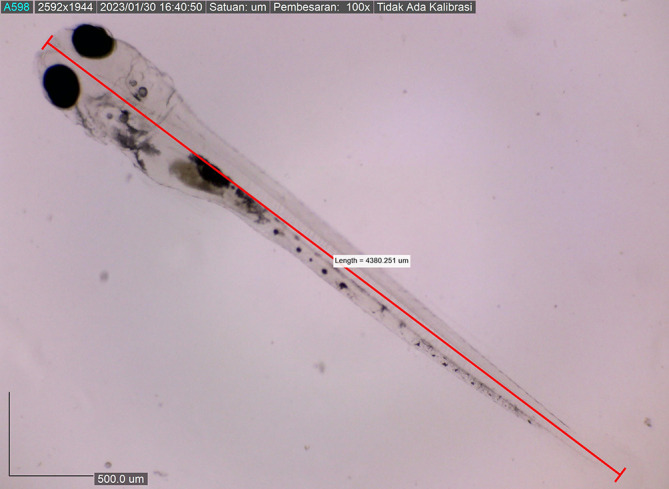
Sixth day: larva with a stomach filled with artificial feed (stomach is light grey), total larval length was 4380.251 ± 0.0025 μm.

**Figure 12.**
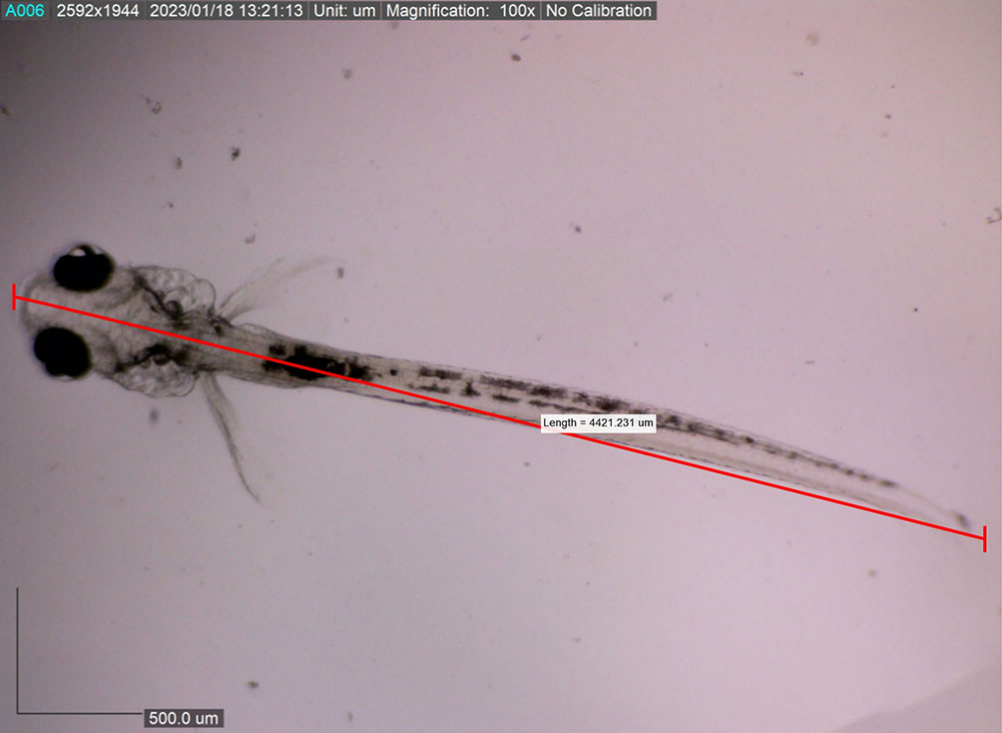
Eight-day-old post-hatch larva showing the appearance of the dorsal, caudal, and pelvic fins (4421.231 ± 0.0028 μm in total length).

Larval body length reached 4380.251 ± 0.0025 μm by the eighth DPH; by this time, the amount of BP Eguchi was increased to satisfy the consumption need. The amount of feed consumed was more observable in the stomach ( Figure 11). The body length of the larvae reached approximately 4421.231 ± 0.0028 μm on the eighth DPH; at this stage, the dorsal and pelvic fins began to appear ( Figure 12). We gave them more artificial feed to increase survival and growth, which could be seen spreading in the stomach (the feed is black).

**Figure 13.**
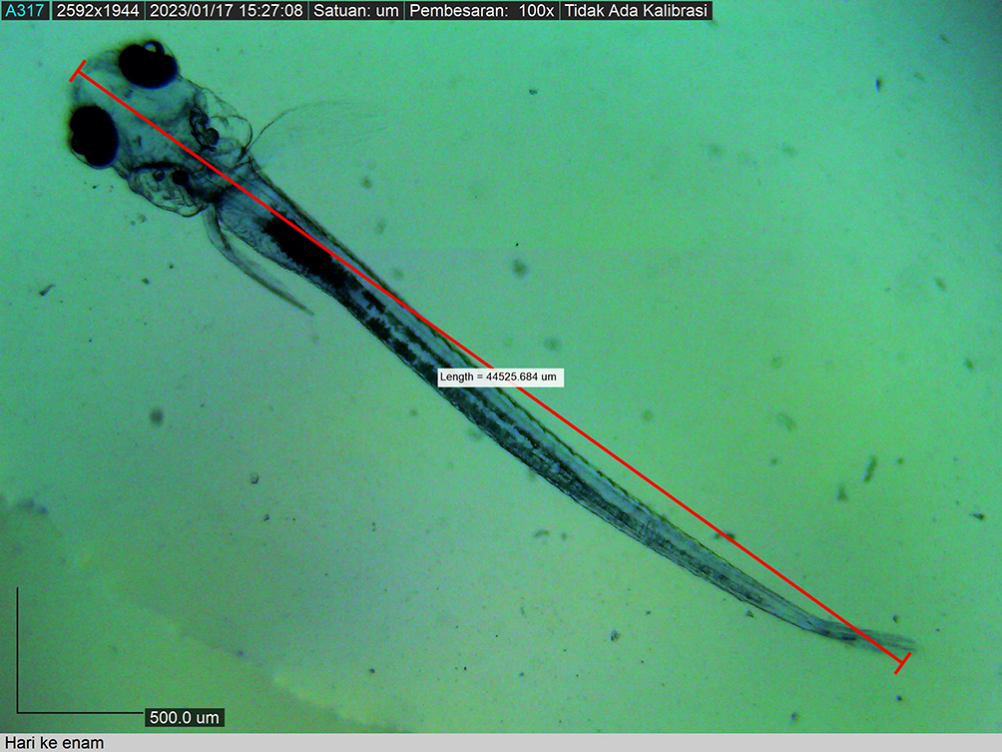
Twelve-day post-hatch larva showing the appearance of the dorsal, caudal, and pelvic fins (5850.2332 ± 0.0025 μm in total length).

**Figure 14.**
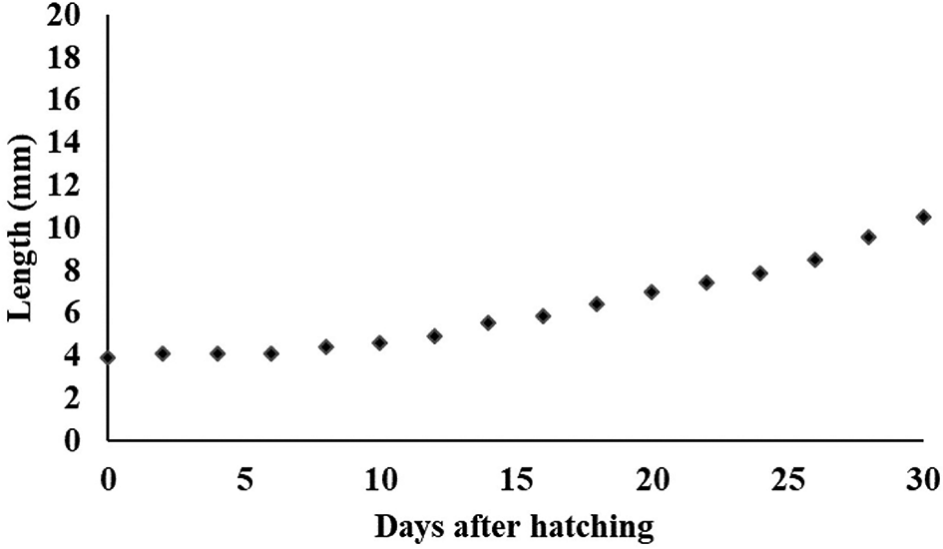
Growth of *Mystacoleucus padangensis* larvae reared over 30 days.

**Figure 15.**
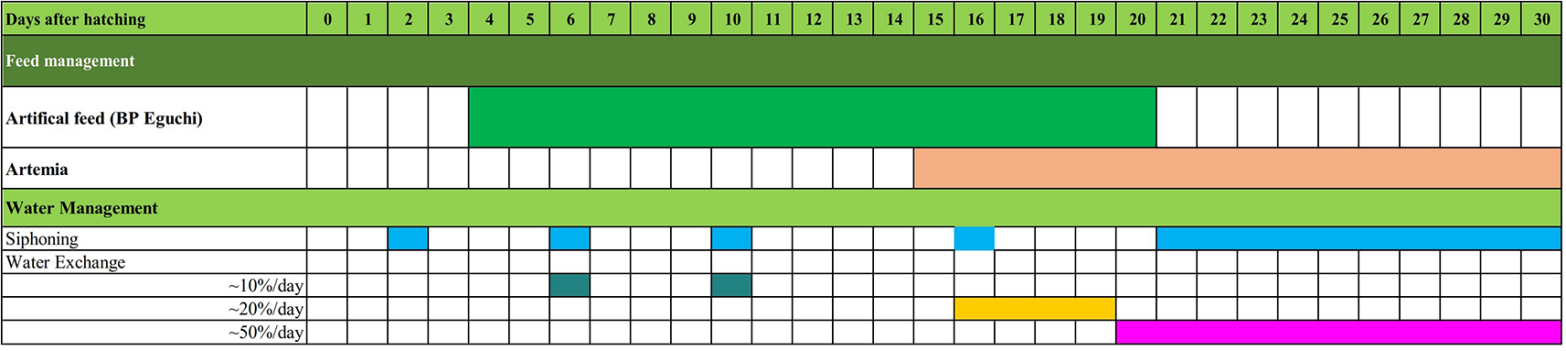
A systematic food and water management protocol for rearing *M. padangensis* larvae.

The larvae grew to 44525.684 ± 0.0019 μm by the 12 ^th^ DPH, by which time all fin types were well demarcated. Chromatophore pigmentation started during embryo development and became intense as the larvae grew. The larval body colour was transparent until the 12 ^th^ DPH ( Figure 13). Live food ( *Artemia nauplii*) was fed to the larvae from the 17 ^th^ DPH when larval body length reached approximately 5850.2332 ± 0.0025 μm. Larvae started metamorphosis by the 15 ^th^ DPH when their body length had reached 6450.0935 ± 0.0457 μm and was completed by the 22nd DPH when their body length had reached 7430.27 ± 0.0638 μm. The larval body remained transparent until the completion of metamorphosis into juveniles ( Figure 13). At 30 DPH larvae rearing, the body length reached 10500.155 ± 0.06 μm ( Figure 14), and the survival rate was 28.4 ± 3.04%, with feed and water management protocols as summarised in Figure 15. This fact indicates the first successful complete metamorphosis in *M. padangensis.*

## Discussion

### Reproduction characteristics

*M. padangensis* is a potential candidate species for freshwater aquaculture because it has a high market price but is threatened by overfishing. ^22^ This study is the first to detail the reproductive characteristics of broodstock reared in ponds for preliminary spawning and larval rearing of *M. padangensis* in hatcheries. *Mystacoleucus padangensis* is classified as a small indigenous species that is essential for consumers as a food source. This species has successfully been artificially bred at the female broodstock measuring 7.56 and 9.31 cm, and the male broodstock measuring 4.86 and 6.38 cm.

Our study showed that the body weight of female *M. padangensis* fish reared in ponds before spawning varied from 5.85 to 9.38 g/fish, with a GSI ranging from 9.78 to 16.95%. This is smaller than *M. padangensis* broodfish caught from the wild, each of which weighs 10 to 15 g with a GSI of 13.09 to 22.36%. ^33^ The fecundity of broodfish reared in ponds ranged from 3,539 to 5,656 eggs/fish. In the wild, it varies from 3,469 to 6,531 eggs/fish. ^21^ The female weight of broodstock and absolute fecundity had a strong relationship ( *r* ^2^ = 95%, *P* = 0.000). The differences in absolute fecundity in maturing broodfish depend on the brood weight, age of broodfish, and species, including strain. ^6^^,^^34^^–^^36^ Female *M. padangensis* broodfish oocyte development is of the synchronous batch type. One bag of eggs will have up to 80% spawning, with sizes ranging from 374 to 558 μm. This condition is related to the spawning behaviour of *M. padangensis* in the wild: the fish migrates to rivers with shallow water characteristics (water depth 0.41 m), bottom substrate consisting of sand, gravel, and caracal, and a current speed of 47.00 m/sec. ^26^ Conversely, the Indian pompano, *Trachinotus mookalee,* is a pelagic species that inhabits the shallow Indian Ocean and has synchronous batch-type development, ^37^ as does Plata pompano, *Trachinotus marginatus*. ^38^

### Fertilisation and hatching rates

This study showed that the dominant egg diameter for spawning ranged from 466-558 μm. After ovulation, there was an increase in egg diameter ranging from 957.941-1180.156 μm, equivalent to 50.30-52.70%. Previous studies showed there was an increase of 27.8-32.14% ^6^ in the egg diameter of the giant gourami ( *Osphronemus goramy*), an increase of 4.64-32.36% ^17^ in that of the Asian catfish ( *Hemibagrus wyckii*), and an increase of 5.3-27.1% ^39^ in that of the Alakir trout ( *Salmo kottelati*). In the present study, the average fertilisation and hatching rates at a water temperature of 24-26 °C were 85.93 ± 3.07% and 78.93 ± 4.13%, respectively. Fertilisation rates and hatching rates vary between candidate species for aquaculture, *e.g.*, 69 ± 1.55% and 87.67 ± 0.81% for *Trachinotus mookalee*, ^37^ 60.91 ± 4.68% and 42.91 ± 2.92% for *Hemibagrus wyckii*, ^17^ 81.60 ± 3.37% and 76.40 ± 2.22% for *Osphronemus goramy*, ^6^ and 88.3 ± 2.88% and 65.02 ± 7.02% for *Salmo kottelati*. ^39^ Differences in fertility and hatching rates can be caused by the quality of sperm, ^40^^,^^41^ temperature of the water in the hatching tank, activating medium, ^42^^–^^44^ egg stocking density, ^45^^,^^46^ and heavy metal toxicity. ^47^

### Larval rearing

The development of larval morphology for each fish species is almost the same, but the initial growth of the larvae is different. In this study, the specific growth rate of *M. padangensis* larvae was estimated at 3.30% per day over 30 days of larval rearing. The length of the newly hatched larvae was 3.9 mm, and it increased to 10.5 mm over the 30 days after hatching ( Figure 14). In contrast, the growth rate of the Indian pompano ( *Trachinotus mookalee*, Carangidae) from 1 to 30 DPH was 11.4% per day, ^37^ while the growth rate of catfish ( *Clarias magur*) larvae in the different tank colours from 4 to 32 DPH varied from 11.38-11.99%. ^48^ The slower growth of *M. padangensis* larvae was probably caused by the type of feed given during the 30 days of maintenance, starting with artificial feed (BP Eguchi) suitable for the mouth opening of the larvae and continuing with live food, such as *Artemia nauplii*. Some researchers have suggested that commercial feed for larvae has undergone considerable advancement. However, live feeds such as *Artemia nauplii*, *Daphnia manga*, rotifers, copepods, among others, are still necessary for most larvae at feeding times. ^49^^–^^51^ According to Tancioni *et al.*, ^52^ the growth rate of the brook chup ( *Squalius cutemonis,* Cyprinid) when fed rotifers between 10 and 20 DPH is 0.8%/day; when fed copepods (from 40-50 DPH), the growth rate slightly increases to 2.4%/day. The different feeding regimes have evident effects on larval performance. Larvae Ballan wrasse ( *Labrus bergylta*) receiving copepods as their initial diet indicated significantly higher survival rates than those fed on rotifers. ^50^ Whether *M. padangensis* larvae receiving rotifers as an initial diet, continuing with copepods, and so on with *Artemia nauplii* can increase larval survival by more than 28.4 ± 3.04% is still poorly understood. In addition to the type of feed given, the stocking density of the larvae (15-300 larvae/m ^-2^) has also been shown to affect the growth of giant gourami ( *Osphronemus goramy*) larvae reared from 7 DPH to 21 DPH. ^53^ In addition, for tambaqui ( *Colossoma macropomum*) larviculture, the best stocking density is 180 larvae/L. ^54^ In the present study, whether the growth rate of larval *M. padangensis* was affected by food type and stocking density was not clear. Therefore, a clear nutritional protocol and optimal stocking density to enhance the growth of *M. padangensis* larvae are necessary for future analyses.

In this study, with 30 days of larval rearing with the feeding protocol, illustrated in Figure 15, the survival rate reached 28.4 ± 3.04%, which was the first success in *M. padangensis* aquaculture. Similarly, the average survival rate of 21.53 ± 1.45% for Indian pompano larvae, *Trachinotus mookalee*, over 25 days. Researchers have reported that the survival rate for the larvae of each fish species varies depending on the feeding protocol. ^37^^,^^50^^,^^52^ Additionally, poor nutrition during larval development has an adverse effect on survival rates. Malnutrition causes a decrease in larval survival, for example, by indirectly decelerating the growth rate, resulting in a longer larval stage and poorer larval activity ability, increasing predators’ opportunities to prey on the larvae. ^55^^–^^57^ The diverse feeding regimes have evident consequences on larval development. Larvae receiving rotifers as their initial diet show significantly lower survival rates than those fed copepods. ^50^ Larval survival is influenced not only by feeding protocols and poor nutrition, but also by rearing tank background colour, ^48^ water temperature, ^37^^,^^58^^,^^59^ different stocking densities, ^53^^,^^60^ and live food in aquaculture. ^61^^–^^63^ All these factors are challenges to increasing the survival and growth of *M. padangensis* larvae.

## Conclusions

This study shows that *M. padangensis* can be domesticated under farming conditions by broodstock development, which leads to final gonadal maturity, namely the reproductive organs (gonads) of the broodfish, both male and female, have reached the level of full sexual maturity. This report is the first successful record of wild fish gonadal maturation under farming conditions, induced spawning, and larval rearing of this species with an indoor hatchery system. The maturing broodstock in captivity can be used for seed production by artificial spawning. The high hatchability of eggs ranges from 77 to 86%, and the low larval survival rate of 28.4% creates challenges that must be resolved in larval rearing, such as feeding protocols, types of feed used, live food in larval rearing, larval stocking density, water quality, including the background colour of the larval rearing tank to make *Mystacoleucus padangensis* an excellent candidate for aquaculture.

## Data Availability

Figshare: Broodstock development, induced spawning and larva rearing of the bilih, Mystacoleucus padangensis (Bleeker, 1852), a vulnerable species, and its domestication potential for new candidate aquaculture., https://doi.org/10.6084/m9.figshare.22179500.v4. ^64^ This project contains the following underlying data:
-
Table 1. Raw data female size and characteristics reproduction -
Table 2. Raw data male size and sperm characteristics of *M. padangensis* on the farm -
Table 3. Raw data Distribution of egg diameters in each of gonadal maturity stages II, III, and IV -
Table 4. Raw data fertilization rate and hatching rate of *M.padangensis*
-
Table 5. Raw data larva development newly hatched to 30th DPH (μm) Figshare: ARRIVE Essential 10 checklist for “Broodstock development, induced spawning and larval rearing of the bilih, *Mystacoleucus padangensis* (Bleeker, 1852), a vulnerable species, and its potential as a new aquaculture candidate, https://doi.org/10.6084/m9.figshare.22179500.v4. ^64^ Data are available under the terms of the Creative Commons Attribution 4.0 International License (CC-BY 4.0).
