# Supplementary material for: Broodstock development, induced spawning and larval rearing of the bilih, Mystacoleucus padangensis (Bleeker, 1852), a vulnerable species, and its potential as a new aquaculture candidate
Source: F1000Res. 2023 Oct 26;12:420. Originally published 2023 Apr 19. [Version 2] doi: 10.12688/f1000research.132013.2 (PMC10624952; doi:10.12688/f1000research.132013.2)
Supplement: Peer review history

# Reviewer report: Reviewer response for version 2

**DOI:** 10.5256/f1000research.157814.r218331

**Published:** 2023-11-03

**Authors:** Safaa M Sharaf

**Affiliations:** 1 Animal Prod. & Fish Resources, Suez Canal University, Ismailia, Egypt

The authors had made a number of significant changes to this paper. With these changes, the paper becomes more comprehensive and provides readers with clearer and more detailed information.

Is the work clearly and accurately presented and does it cite the current literature?

Yes

If applicable, is the statistical analysis and its interpretation appropriate?

Partly

Are all the source data underlying the results available to ensure full reproducibility?

Yes

Is the study design appropriate and is the work technically sound?

Yes

Are the conclusions drawn adequately supported by the results?

Partly

Are sufficient details of methods and analysis provided to allow replication by others?

Partly

Reviewer Expertise:

Fish biology

I confirm that I have read this submission and believe that I have an appropriate level of expertise to confirm that it is of an acceptable scientific standard.

# Reviewer report: Reviewer response for version 2

**DOI:** 10.5256/f1000research.157814.r218332

**Published:** 2023-10-27

**Authors:** Md. Moshiur Rahman

**Affiliations:** 1 Department of Biology and Agricultural Engineering, University of California Davis, Davis, California, USA

It looks perfect. Thanks.

Is the work clearly and accurately presented and does it cite the current literature?

Partly

If applicable, is the statistical analysis and its interpretation appropriate?

Partly

Are all the source data underlying the results available to ensure full reproducibility?

Yes

Is the study design appropriate and is the work technically sound?

Yes

Are the conclusions drawn adequately supported by the results?

Yes

Are sufficient details of methods and analysis provided to allow replication by others?

Partly

Reviewer Expertise:

Fish reproduction, larval rearing, sperm quality assessments, fish biology, and fish nutrition.

I confirm that I have read this submission and believe that I have an appropriate level of expertise to confirm that it is of an acceptable scientific standard.

# Reviewer report: Reviewer response for version 1

**DOI:** 10.5256/f1000research.144909.r197812

**Published:** 2023-10-17

**Authors:** Safaa M Sharaf

**Affiliations:** 1 Animal Prod. & Fish Resources, Suez Canal University, Ismailia, Egypt

Broodstock development, induced spawning and larval rearing of the bilih, *Mystacoleucus padangensis* (Bleeker, 1852), a vulnerable species, and its potential as a new aquaculture candidate, is an important research study of *Mystacoleucus padangensis *living in Lake Singkarak, Indonesia, which has high potential market demand but is threatened by overfishing and has not been successfully cultured. I appreciate the authors' efforts in conducting this research. However, I have specific comments on some sections, which I have provided below:

 Is the journal system to mention only the methods without the materials in the research?

**Methods:**

- The authors used clove oil, 5 ml/10 liters, and did not mention for how long (minutes).
- The authors did not mention the injected dose of gonadotropins and ovaprim/fish only the injection volume was mentioned as 0.1 ml/fish.
- The authors did not mention in the methods that the growth of larvae will be calculated during the 30 days, and this period is too short to estimate the growth of fish. Besides, the length gain is not mentioned as a growth performance parameter.
- Please in the methods, you must mention how these measurements were done all the analysis of CF, GSI, AF, RF (relative fecundity measured for length or for weight?), MOL and its formula or equations with a reference.
- Page 4: "the oocyte nucleus was classified using a four-stage scale as follows Stage 1: germinal vesicle in the central position, stage 2: early migration of the germinal vesicle (less than half of radius), stage 3: late migration of the germinal vesicle (more than half of radius), stage 4: germinal vesicle in the periphery or germinal vesicle breakdown (GVBD)." - Are these authors (Ref. 24, Krejszeff S, Katarzyna T, Daniel Z, *et al.*) the original ones who published this classification? It is preferable to write in methods the original author.

**Results:**

- Does the size of the eggs mean the diameter of the eggs? Page 5 and in the Figure 3? If yes, scientifically, it is better to mention the egg diameter, not the size of the egg.
- The shape of the yolk sac was not identified or marked in the figures 6, 7 and 8 as it was determined in the presence of artificial food at Figure 10.
- Figure 5 - What type of stage of larval development is this? It is better to mention it and mention its reference.
- The title under the figures is preferable to mention the length of 6-day-old post hatch larvae showing..., and so on in all the figures, from Figure 6 to 13
- Is this fish species (4-7g) required by the consumer as eatable or as an ornamental fish? Because of its very small size, is it worth it to be artificially hatched?

**Conclusions**

- The survival rate of 28.4% is very low not as mentioned in the conclusion ( **Conclusions: **Its successful spawning induction and high larval hatching and survival rates make *M. padangensis *an excellent aquaculture candidate). The reasons must be known by doing another future research because the fertilization and hatching rates of *M. padangensis* were high and promising.

Is the work clearly and accurately presented and does it cite the current literature?

Yes

If applicable, is the statistical analysis and its interpretation appropriate?

Partly

Are all the source data underlying the results available to ensure full reproducibility?

Yes

Is the study design appropriate and is the work technically sound?

Yes

Are the conclusions drawn adequately supported by the results?

Partly

Are sufficient details of methods and analysis provided to allow replication by others?

Partly

Reviewer Expertise:

Fish biology

I confirm that I have read this submission and believe that I have an appropriate level of expertise to confirm that it is of an acceptable scientific standard, however I have significant reservations, as outlined above.

# Reviewer report: Reviewer response for version 1

**DOI:** 10.5256/f1000research.144909.r170203

**Published:** 2023-05-04

**Authors:** Md. Moshiur Rahman

**Affiliations:** 1 Department of Biology and Agricultural Engineering, University of California Davis, Davis, California, USA

The authors have conducted an important research study on broodstock development, induced spawning, and larval rearing of the bilih, *Mystacoleucus padangensis* (Bleeker, 1852), a vulnerable species with potential as a new aquaculture candidate. I appreciate the authors' efforts in conducting this research. However, I do have specific comments on each section, which I have provided below:

**Comments:**

**Abstract: **

- Background: Please add "yet" - " *has not* **yet** *been successfully culture*d."
- Methods: " *The broodfish were fed commercial feed to satiation at 09:00 and 17:00 h*." How many times?
- Results: The mouth opened at 72 DPH? Please write the elaborated form of DPH as it was written for the first time in order to better understand the context in which it was used.
- Results: *Artemia* is a genus of brine shrimp. The standard convention for scientific writing is to italicize the names of genus and species, including *Artemia*. Therefore, it is recommended to write *Artemia* in italic font in scientific or academic papers.
- Results: " *Weaning of larvae started at 4 DPH*." How did you start - co-fed with *Artemia* or directly feeding with dry feed?
- Conclusion: I’d suggest rephrasing this paragraph such as: The successful induction of spawning, as well as high rates of larval hatching and survival, make *M. padangensis* a highly desirable candidate for aquaculture.

**Introduction: **

- 2 ^nd^ paragraph: Please do not start it with " *Therefore*" and rephrase it.
- 3 ^rd^ para: "Nile tilapia" is not the scientific name of a species. It is a common name that refers to several different species of tilapia, which belong to the family Cichlidae. So, please do not italicize it.
- 4 ^th^ para: I can see a big jump here before introducing *Mystacoleucus padangensis*. Please make a perfect link to introduce it. For example, before discussing this species, which is a freshwater fish species native to the rivers of Sumatra, it is important to understand the context of its ecological niche within the aquatic ecosystem.
- 5 ^th^ para: I can see that it is a small fish. However, why does it have a high market demand? Could you provide specific references to support this claim?

**Methods:  **

- Could you please rephrase the sentence as follows: " *They were adapted and reared to oocyte development under farming conditions.*" **The correct sentence is: "During the oocyte maturation stage of development..."
- " *Feed was given to satiation daily at 09:00 and 17:00 h*." How many times, ration, amount, etc.?
- May I know if the fish length (FL) was measured as total length, standard length, or fork length?
- " *Broodfish were analysed for the condition factor (CF), gonadal weight (GI), gonadal somatic index (GSI), absolute fecundity (AF), relative fecundity (RF), egg diameter (ED), semen volume (SM), semen pH (SpH), and motility of spermatozoa (MOL) from male broodfish.*" Please write briefly how and which techniques (also provide appropriate references for each one) were followed to analyze them?
- " *The average fish weight (FW) and fish length (FL) of the twenty female broodfish were 7.56 ± 0.85 g and 9.31 ± 1.36 cm, respectively; those of the twenty male broodfish were 4.86 ± 1.20 g and 6.38 ± 0.30 cm, respectively.*" Please split these sentences to understand them better.
- " *The relationships between female brood weight and absolute fecundity and gonadal weight, as for relationships between male sow weight and gonadal weight and semen volume, were assessed using the least square’s regression method.*" Also split these sentences too.

**Results: **

- " *Mystacoleucus padangensis*" must be italicized.
- In the 'Methods' section, it is necessary to provide a brief explanation of how you measured or analyzed reproductive characteristics such as weight, GSI, fecundity, and others.
- Also, please define and show how you estimated the absolute fecundity in the 'Methods' section.
- Please split the sentences providing the outcomes of the relationship to understand them better.
- What does * mean in the equation?
- Prior to conducting the regression analyses, did you verify that the assumptions were met? As fecundity is a count variable, it is likely that it may not follow a normal distribution. Please provide clearly this information in the statistical section.
- As previously stated, it is important to clarify in the tables whether FL refers to total length, fork length, or standard length.
- Is a sample size of n=20 sufficient to yield meaningful results? Please do a power analysis or provide the effect size for these outcomes.
- Please do an individual analysis to explore the variation among and/or between different GMS (shown in Fig. 2), fertilization rate, and hatching rate (shown in Fig. 4).

**Discussion: **

- " *a potential candidate species for freshwater aquaculture because it has a high market price*" - Please explain why with appropriate references.
- Overall, the discussion section appears to be inadequately written. The comparison of each finding with others lacks relevance and clarity. It is important to provide a clear explanation of why these findings should be compared and discussed with other studies and findings, and to provide possible explanations.

**Conclusion: **

- What do you mean by " *final gonadal maturity*"?

Is the work clearly and accurately presented and does it cite the current literature?

Partly

If applicable, is the statistical analysis and its interpretation appropriate?

Partly

Are all the source data underlying the results available to ensure full reproducibility?

Yes

Is the study design appropriate and is the work technically sound?

Yes

Are the conclusions drawn adequately supported by the results?

Yes

Are sufficient details of methods and analysis provided to allow replication by others?

Partly

Reviewer Expertise:

Fish reproduction, larval rearing, sperm quality assessments, fish biology, and fish nutrition.

I confirm that I have read this submission and believe that I have an appropriate level of expertise to confirm that it is of an acceptable scientific standard, however I have significant reservations, as outlined above.
